# Rigid External Distractors in Midface Fractures: A Review of Relevant and Related Literature

**Published:** 2020-10-19

**Authors:** Zachary Gala, Jordan Halsey, Samuel Kogan, Ian Hoppe, Frank S. Ciminello, Mark S. Granick

**Affiliations:** ^a^Division of Plastic and Reconstructive Surgery, Rutgers New Jersey Medical School, Newark, NJ; ^b^Division of Plastic and Reconstructive Surgery, Rutgers Robert Wood Johnson Medical School, Piscataway, NJ; ^c^Division of Plastic and Reconstructive Surgery, University of Mississippi Medical Center, Jackson, MS; ^d^Division of Plastic and Reconstructive Surgery, Hackensack University Medical Center, Hackensack, NJ

**Keywords:** rigid external distraction, RED, midface fractures, facial trauma, panfacial injury

## Abstract

Introduction: Literature discussing the use of rigid external distraction devices in midfacial trauma is limited. Rigid external distraction devices have been described for use in craniofacial surgery, allowing for distraction and stabilization of bony segments. In complex facial trauma, bony fragments are often comminuted and unstable, making traditional approaches with internal fixation difficult. Moreover, these approaches require subperiosteal dissection, limiting blood supply that is important for bone healing. Objective: The goal of this study was to evaluate the role of rigid external distraction devices for the treatment of complex facial trauma. Methods: We performed a literature review of rigid external distraction devices, as relevant both for facial trauma and for other craniofacial indications, to better elucidate their use and efficacy in complex facial fractures. Results: The review revealed only 2 articles explicitly describing rigid external distraction devices for facial trauma, while 6 other articles describing its use for other craniofacial cases. An important benefit associated with the use of rigid external distraction devices is their ability to provide controlled traction of bony segments while also allowing for movement as needed for fracture reduction. Various articles describe performing internal fixation following rigid external distraction device usage, while others emphasize that internal fixation is not necessarily indicated if the rigid external distraction device is left intact long enough to ensure bony healing. One potential setback described is unfamiliarity with using the rigid external distraction device, which can preclude its use by many surgeons. In addition, the literature review did not provide any uniform guidelines or recommendations about how long rigid external distraction devices should remain intact. Conclusion: Based on relevant literature, rigid external distraction devices have been shown to be useful in the stabilization and treatment of complex facial fractures. Further studies should be conducted to better elucidate the specific indications for rigid external distraction devices in complex facial trauma.

## Summary of Review:

Literature discussing the use of distraction ostogenesis (DO) and rigid external distraction (RED) devices in midfacial trauma is sparse. Our study seeks to analyze the available literature on these techniques, specifically as they are used in trauma rather than their traditional use in craniofacial surgery.

Distraction osteogenesis (DO) is the process by which bone is cut and separated, allowing for osteogenesis to occur. First proposed by Von Langenbeck in 1869 and first reported clinically by Cadivalla in 1905,^1^ DO was historically utilized to correct lower extremity length discrepancies. Later, Ilizarov popularized the use of DO, describing the use of an external device to hold bony fracture segments in traction for osteogenesis to occur within the gap.^1^ Later, its principles were applied to craniofacial surgery under McCarthy et al.^2^ Now, this technique is employed in a wide variety of craniofacial clinical conditions, such as hemifacial microsomia, craniosynostosis, and micro/retrognathism.^1^ In addition, rigid external distraction (RED) devices are used to assist with complex orthognathic and midface procedures.

RED devices have been described for use in severe facial fractures. However, there is a paucity of literature on the use of these RED devices, specifically for midfacial trauma. Often, facial fractures are treated with internal fixation using plates and screws. This technique requires extensive dissection, with plates placed in a subperiosteal plane. In severely comminuted fractures, this would eliminate the remaining blood supply, leading to bony resorption. Furthermore, in complex and comminuted fractures, there can be difficulty reducing fractured segments. RED devices do not violate the periosteum and could be useful in the treatment of severely comminuted, unstable injuries.

Our study reviews the available literature citing the use of RED devices in midfacial trauma in order to provide a framework for the indications and use of RED devices in these cases.

## METHODS

The authors searched the available literature using key words, including but not limited to, “Rigid External Distraction,” “RED,” “Distraction Osteogenesis,” “DO,” “Midfacial fractures,” “Trauma,” and “Facial Fractures.” Because of the paucity of literature, the authors decided against using typical “inclusion” and “exclusion” criteria. Instead, articles were categorized as either “relevant” (articles that consisted of all cases of midface fractures with resultant use of RED devices) or “related” (articles that consisted of non–midface fractures such as mandible fractures, nontraumatic causes, or had no mention of the use of RED and/or DO). This allowed for the “related” articles to be used for comparative analysis in the discussion of this review.

## RESULTS

The literature review yielded 8 articles. Two articles were classified as “relevant”: one case report (N = 1) and one case series (N = 6). The case report described the use of RED devices for panfacial fractures, whereas the case series outlined 6 patients with traumatic facial fractures and the subsequent use of RED devices.

The remaining 6 articles that were classified as “related” consisted of 5 case series (N = 8, 7, 19, 8) and 2 case reports (N = 1, 1). Four of the case series followed patients with nontraumatic causes, commonly midface hypoplasia and/or craniosynostosis. One case report presented deep vein thrombosis (DVT) as a complication of RED use, and the other case report outlined the use of a RED device in an adult with Crouzon syndrome. Table 1 provides more details about the articles included in this literature review.

## DISCUSSION

There is a paucity of literature on the use of RED devices in the treatment of midface fractures. To our knowledge, this is the first literature review evaluating this treatment method for use in facial trauma.

There were 2 “relevant” articles. A case report by Hihara et al^3^ describes the use of a novel RED device for panfacial fractures of traumatic cause. The patient's injuries included bilateral zygoma fractures, naso-orbitoethmoid (NOE) fracture, open mandibular condyle fracture, and comminuted mandibular body fractures. Operative intervention was delayed because of uncontrolled hemorrhage. A RED device was placed 4 weeks after initial presentation and remained in place for 8 weeks. The authors noted that internal fixation with plating would have required subperiosteal dissection and thus increase the risk of avascular necrosis and resorption due to the significant comminution of the fracture fragments.^3^ The RED device avoided the need for subperiosteal disruption. Moreover, the RED system provided a minimally invasive, low-cost, and versatile method of fixation to obtain occlusion. Of note, the authors did not implement subsequent internal fixation.

A case series by Canter et al^4^ followed 6 patients with complex facial fractures including NOE, zygomaticomaxillary, LeFort II, and mandible fractures. All of the patients tolerated RED use with no complications. Some patients underwent a combination of both internal fixation and the RED device. The duration of RED use averaged 2 weeks. The reasons cited for use of RED devices in these trauma cases were similar to those of Hihara et al.^3^ The authors noted that patients exhibited acceptable aesthetic and functional results throughout follow-up, showing controlled traction of impacted bony segments.^4^ Of note, the RED system successfully overcame soft-tissue tension, enabling the use of smaller plates for internal fixation, while showing the additional advantage of allowing for fine adjustments postoperatively without the need for further invasive intervention.^4^

The other 6 articles were classified as “relevant,” as they did not directly pertain to the use of RED devices for midface fractures but did involve the use of RED devices for nontraumatic causes such as midface hypoplasia and craniosynostosis. These studies are important to analyze in order to better understand the general indications and recommendations surrounding the use of RED devices, especially since the literature on the use of these devices for midface fractures is scarce.

A case series by Iannetti et al^5^ followed 8 patients with RED devices used for LeFort III advancement. This series included radiographic analysis of several anatomic landmarks throughout the use of RED devices for advancement, demonstrating that a RED device allows for excellent vector control.^5^ Another case series by Hara et al^6^ investigated the safety and efficacy of a new endoscopic surgical technique in 7 patients with midface hypoplasia and malocclusion. Their technique involved the use of an intraoperative RED system. The authors highlighted that RED usage was safe, reliable, and effective. Furthermore, the RED system made the endoscopic, minimally invasive approach possible. A study by Kanno et al^7^ in 2008 evaluated the degree of advancement and stability of the maxillary segment in 19 patients with maxillary hypoplasia who underwent LeFort advancement. Fourteen patients utilized RED devices, which the authors noted provided the most stable and reliable method of advancement.^7^ Another case series investigated 8 patients with syndromic craniosynostosis to compare RED devices with internal distraction systems for LeFort III advancement.^8^ The authors noted that RED systems were advantageous in that they allowed for controlled traction and negated the need for operative removal of the device.

Roussel et al^9^ presented a case report in which a patient suffered DVT as a complication of RED use. This complication was not described in other articles related to RED use. Wang and Liu^10^ described an adult patient with Crouzon syndrome who underwent LeFort III osteotomy and RED placement. The authors noted that the patient's symptoms of exorbitism and airway compromise were relieved and that the use of the RED system aided in overcoming the soft-tissue pull, resulting in adequate and tolerable traction during distraction.^10^

Analysis of the available literature demonstrated several benefits of using RED devices for complex facial trauma. One benefit is that a RED device can serve as either *fixator* or *distractor* in the treatment setting. Furthermore, distraction allows for 2 important biomechanical phenomena to occur: creep and stress relaxation.^3^^,^^4^ Creep holds segments under constant tension, and stress relaxation allows soft tissues to relax while remaining under constant strain. Rigid distraction allows for controlled traction of impacted bony segments, and by doubling as an external fixator, the surgeon can potentially decrease the number of miniplates used. RED devices can overcome soft-tissue tension, while maintaining the possibility for fine adjustment of bony segments postoperatively.^3^^,^^4^ Moreover, in the event of postoperative infections, there is no need for hardware removal.

**Figure 1:**
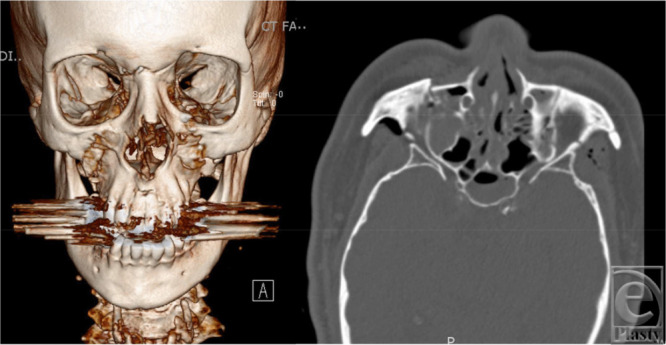
Case example of a patient with panfacial fractures planned for RED device usage. Left: 3-dimensional reconstructed image; Right: axial image. RED indicates rigid external distraction.

**Figure 2:**
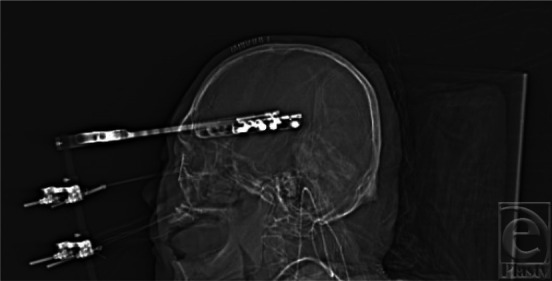
Sagittal image of a patient with RED device intact. RED indicates rigid external distraction.

**Figure 3:**
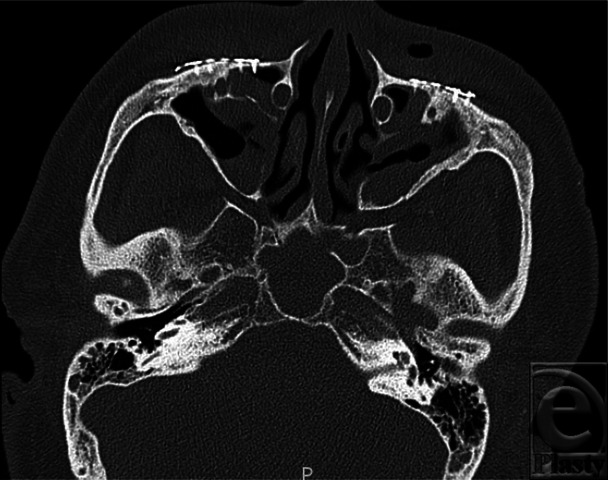
Axial image of a patient following RED device removal with subsequent fixation of facial fractures with titanium miniplates. RED indicates rigid external distraction.

There are some potential drawbacks of utilizing RED devices for midface fractures. RED usage is subject to familiarity and experience with the device. Multiple authors cite extensive prior use and “modifications to simplify usage” in their studies. There is also significant variability in the management, outcomes, and sequelae in these cases. Moreover, patients with severe facial trauma often present with multiple concomitant injuries as a result of high-velocity trauma. These extensive injuries may necessitate the need for other, more emergent operative interventions that could delay or preclude the placement of a RED device. Notably, the articles did not provide a uniform recommendation for how long the RED device should be left intact for adequate bony healing. Figures 1 to 3 demonstrate the use of a RED device in a patient at our institution with panfacial fractures.

Our study has some limitations. The studies varied widely in cause and because the indications for surgery differed, the exact procedures and techniques were not uniform. The paucity of available data and outcomes makes these investigations limited in their generalizability and impede the ability to draw conclusions. Furthermore, the literature review showed the lack of available data on the use, management, outcomes, and complications of this treatment.

To summarize, the authors present an alternative technique for the treatment of complex midface fractures that could potentially be used rather than the traditional method of open reduction and internal fixation (ORIF) in special circumstances. It is neither necessary nor indicated for all midface fractures; however, RED devices may be indicated for complex fracture patterns with severe comminution, significant periosteal stripping resulting in compromised bone vascularity, and nonideal surgical candidates. Furthermore, a RED device may be indicated in situations when fracture fixation is performed as more of a temporizing measure prior to internal fixation, as in some patients with other critical injuries. While the authors present no absolute contraindications to RED use, inexperience/unfamiliarity with RED systems may lead the surgeon toward traditional ORIF. Further studies should be conducted to better elucidate the efficacy of RED devices in complex facial trauma.

## Figures and Tables

**Table 1:** Articles included in RED device literature review*

	Article title	Author	Article type	Number of patients	Article summary
Relevant	A novel fixation method for panfacial fracture using an Ilizarov-type external fixator	Hihara et al^3^	Case report	1	Use of a novel RED device for panfacial fractures; RED use avoided need for subperiosteal dissection, reducing the risk of avascular necrosis and infection.
Relevant	Use of rigid external distraction device in treatment of complex maxillofacial fractures	Canter et al^4^	Case series	6	Described RED use for patients with traumatic facial fractures. All tolerated the RED device with no complications. RED use exhibited controlled traction, overcame soft-tissue pull, and allowed for small postoperative adjustments without need for operative intervention.
Related	Le Fort III external midface distraction: surgical outcomes and skeletal stability	Iannetti et al^5^	Case series	8	A RED device was used after LeFort III osteotomy and midface advancement. RED use allowed for better vector control, resulting in more precise and effective distraction.
Related	Endoscopically assisted intraoral modified Le Fort II type midfacial advancement using piezoelectric surgery and an intraoperative RED system	Hara et al^6^	Case series	7	Investigated safety and efficacy of a new endoscopic surgical approach, intraoral vs traditional coronal flap for patients with midface hypoplasia and malocclusion; RED use was shown to be safe, reliable, and effective.
Related	Long-term skeletal stability after maxillary advancement with distraction osteogenesis in nongrowing patients	Kanno et al^7^	Case series	19	Measured degree of skeletal advancement and stability in patients with maxillary hypoplasia; RED use provided the most stable and reliable method of advancement.
Related	Midface distraction following Le Fort III and monobloc osteotomies: problems and solutions	Gosain et al^8^	Case series	8	Compared RED devices with internal distraction systems for patients with syndromic craniosynostosis. RED systems allowed for more controlled traction and negated the need for an additional operative intervention to remove the device.
Related	Deep venous thrombosis in teens with Crouzon syndrome post-Le Fort III osteotomy with rigid external distraction	Roussel et al^9^	Case report	1	DVT is described as a complication of RED use.
Related	Rigid external distractor aided conventional Le Fort III osteotomy advancement in adult with severe midfacial hypoplasia	Wang and Liu^10^	Case report	1	RED use after LeFort III osteotomy in an adult patient with Crouzon syndrome; RED use overcame soft-tissue pull, which resulted in tolerable traction, and reduced possibility of potential relapse.

*RED indicates rigid external distraction; DVT, deep vein thrombosis.
